# Supplementary material for: Understanding Contextual Factors Effects and Their Implications for Italian Physiotherapists: Findings from a National Cross-Sectional Study
Source: Healthcare (Basel). 2021 Jun 7;9(6):689. doi: 10.3390/healthcare9060689 (PMC8226546; doi:10.3390/healthcare9060689)
Supplement: Supplementary file 1 [file healthcare-09-00689-s001.zip › healthcare-1227166-supplementary.pdf]

CONOSCENZA E GESTIONE DEI FATTORI CONTESTUALI: PRESENTAZIONE E PREMESSA METODOLOGICA

Sezione 1/9

Caro collega Fisioterapista,

è attualmente noto che qualsiasi intervento sanitario è dato da una componente attiva/biologica e da una contestuale/psicosociale. È nostro interesse comprendere come, nel panorama italiano fisioterapico, tali fattori contestuali (es. dinamiche interpersonali, setting terapeutico, esperienze e aspettative del paziente) prendono parte all'incontro terapeutico e come vengono gestiti.

Nel caso Lei decidesse di partecipare, Le chiederemo di compilare il questionario della durata di circa 7 minuti. Non ci sono risposte giuste o sbagliate, ci interessa solo sapere il suo punto di vista.

GRAZIE del tempo che vorrà concederci.

Alessandra Bianco e Valentina Capurso,

studentesse del Master in Scienza e Pratica in Fisioterapia Muscoloscheletrica dell'Università degli Studi del Molise

Tutti i dati raccolti saranno trattati e archiviati in maniera rigorosamente anonima ai sensi del D. Lgs. 196/2003 e successive modifiche ed integrazioni in materia di trattamento dei dati personali. A tali informazioni avrà accesso solo il personale coinvolto nello studio. L'accesso a tali dati sarà protetto dallo sperimentatore. La Sua partecipazione Le garantirà la totale riservatezza.

- CONSENSO AL TRATTEMENTO DEI DATI SENSIBILI
- AUTORIZZAZIONE AL TRATTAMENTO DEI DATI SENSIBILI

Sezione 2/9

INFORMAZIONI SOCIO-DEMOGRAFICHE

Genere:

- Maschile
- Femminile
- Altro

Quanti anni hai? (testo risposta breve)

Da quanti anni lavori come fisioterapista? (testo risposta breve)

Qual è il tuo attuale titolo di lavoro?

- Libero professionista
- Dipendente in struttura pubblica
- Dipendente struttura privata
- Docente universitario corso di laurea
- Docente percorso universitario post – graduate (master universitario)
- Docente corsi o percorsi privati post laurea

- Ricercatore
- Altro

Quale campo lavorativo caratterizza prevalentemente la tua pratica clinica?

- Muscoloscheletrico
- Geriatrico
- Neurologico
- Cardio-respiratorio
- Pediatrico
- Sportivo
- Altro

In che regione lavori?

- Abruzzo
- Basilicata
- Calabria
- Campania
- Emilia Romagna
- Friulia Venezia Giulia
- Lazio
- Liguria
- Lombardia
- Marche
- Molise
- Piemonte
- Puglia
- Sardegna
- Sicilia
- Toscana
- Trentino Alto Adige
- Umbria
- Valle d’Aosta
- Veneto

In quale regione hai conseguito il tuo titolo universitario?

- Abruzzo
- Basilicata
- Calabria
- Campania
- Emilia Romagna
- Friulia Venezia Giulia
- Lazio
- Liguria
- Lombardia

- Marche
- Molise
- Piemonte
- Puglia
- Sardegna
- Sicilia
- Toscana
- Trentino Alto Adige
- Umbria
- Valle d'Aosta
- Veneto

Cortesemente, specifica il nome dell'Ateneo (risposta testo breve)

Sezione 3/9

I FATTORI DI CONTESTO

In quale ambiente hai sentito parlare per la prima volta di fattori di contesto?

- Laurea triennale
- Master universitario
- Laurea magistrale
- Corso privato
- Social network
- Questo questionario

Cosa si intende per fattori contestuali? (È possibile selezionare più di una risposta)

- Qualsiasi elemento, anche involontario, con cui il paziente interagisce durante il trattamento
- Strumento terapeutico specifico in grado di influenzare l'esito del trattamento tramite meccanismi neurofisiologici
- Intervento senza un effetto specifico ma con un possibile effetto non specifico
- Strumento diagnostico in grado di discriminare un problema di tipo psicologico da uno tipo organico
- Non saprei definirlo

Quali tra questi ritieni che rappresenti un fattore di contesto: (è possibile selezionare più di una risposta)

- Caratteristiche del paziente (es. credenze, aspettative ecc.)
- Caratteristiche del fisioterapista (onere dovuto, reputazione ecc.)
- Caratteristiche del trattamento (es. scelta della tecnica, approccio incentrato sul paziente ecc.)
- Setting terapeutico (cromatismo, gestione degli spazi ecc.)
- Relazione terapeutica (es. strategie di comunicazione)

A quale dei seguenti fattori di contesto porgi maggiore attenzione nella gestione della tua pratica clinica?

- Caratteristiche del paziente (es. credenze, aspettative ecc.)

- Caratteristiche del fisioterapista (onere dovuto, reputazione ecc.)
- Caratteristiche del trattamento (es. scelta della tecnica ecc.)
- Setting terapeutico (cromatismo, gestione degli spazi ecc.)
- Relazione terapeutica (strategie di comunicazione)

Per quale motivo ritieni maggiormente utile conoscere e saper gestire i fattori di contesto?

- Migliorare la relazione terapeutica
- Migliorare la risposta clinica al trattamento fisioterapico
- Migliorare la soddisfazione percepita dal paziente
- Controllare i sintomi

Con che frequenza utilizzi i fattori di contesto in pratica clinica?

- Mai
- Quasi mai
- A volte
- Spesso
- Sempre

Sezione 4/9

CARATTERISTICHE DEL FISIOTERAPISTA

A quale dei seguenti tuoi aspetti/caratteristiche attribuisci più importanza per potenziare il risultato terapeutico?

- Reputazione professionale
- Uniforme
- Igiene e pulizia
- Strategia comunicative
- Onere

Quale strategia adotti dinanzi alla richiesta da parte del paziente di un trattamento la cui efficacia non è comprovata da evidenze scientifiche disponibili?

- Eroghi la terapia richiesta dal paziente pur consapevole della sua inefficacia;
- Informi il paziente circa l'inappropriatezza della terapia;
- Proponi un trattamento diverso, che rispecchia le tue credenze, conoscenze ed esperienza in merito al quadro clinico
- Proponi l'utilizzo della terapia richiesta, ma solo in seguito al fallimento della terapia precedentemente attuata

Sezione 5/9

CARATTERISTICHE DEL PAZIENTE

Le esperienze pregresse del paziente con altri professionisti sanitari in che modo influenzano la tua strategia terapeutica:

- Eseguo la strategia terapeutica adottata dal precedente professionista sanitario seppur non supportata da evidenze perchè ha dato beneficio al paziente
- Non eseguo la terapia che ritengo più appropriata e specifica al paziente, qualora egli riferisca di averne avuto esperienza negativa in passato
- Le esperienze pregresse non influiscono sulla scelta del trattamento adatto in quel momento
- Assecondo sempre la richiesta del mio paziente

In che modo le aspettative del paziente influenzano la tua strategia di gestione terapeutica:

- Le aspettative del paziente non influenzano la mia scelta del trattamento
- Tengo in considerazione le aspettative del paziente perché esse possono influire sull'esito del trattamento indipendentemente dalla specificità dello stesso
- Cerco sempre di stimolare le aspettative positive allo scopo di potenziare motivazione, alleanza terapeutica e esito clinico
- Cerco di attenuare aspettative negative (basate su lacune informative) illustrando, prima del trattamento, l'efficacia comprovata della strategia scelta

Cambi il tuo modo di approcciarti e/o di impostare il trattamento a seconda dell'età e del genere (uomo, donna, altro) del tuo paziente?

- Si, adatto sempre la pratica clinica di conseguenza
- No, il genere e l'età del paziente non influenza la mia scelta della tipologia di trattamento
- Credo sia importante fare una differenziazione ma non so come adattare la mia pratica clinica
- Mi approccio diversamente a seconda dell'età del mio paziente, ma il genere non credo sia importante

Sezione 6/9

RELAZIONE PAZIENTE-FISIOTERAPISTA

A quale elemento relazionale dai maggiormente importanza nel tuo rapporto con il paziente?

- Aspetto comunicativo
- Disponibilità dinanzi alle richieste del paziente (ES. flessibilità degli orari d'appuntamento)
- Coinvolgimento del paziente nel percorso di cura
- Tocco terapeutico

Qual elemento ritieni maggiormente importante per un'efficiente comunicazione col paziente?

- Ascolto attivo
- Utilizzo di linguaggio tecnico
- Espressioni verbali di supporto e incoraggiamento
- Umorismo e simpatia
- Parafrasi, immagini e metafore per aiutare il paziente a comprendere la sua condizione
- Spiegazione degli effetti e dell'esecuzione del trattamento (overt therapy)
- Coerenza tra linguaggio verbale, paraverbale e non verbale

Sezione 7/9

CARATTERISTICHE DEL TRATTAMENTO

Quali elementi prendi maggiormente in considerazione nel momento in cui organizzi il planning terapeutico?

- Seduta one to one
- Seduta di gruppo per pazienti con problematiche simili
- Seguo la disponibilità del paziente
- L'organizzazione delle sedute dipende dalla condizione clinica del paziente (acuto, subacuto, cronico)

Come gestisci il costo dei servizi da te erogati?

- Le terapie con un brand (marchio) migliore hanno un costo più elevato
- Il costo dipende dal tempo impiegato per la seduta
- Il costo dipende dalla tipologia del trattamento (novità, tecnologia avanzata ecc.)
- Il costo dipende dalla complessità del trattamento
- Il costo dipende dall'esperienza del clinico
- Il costo della seduta è standard

Sezione 8/9

AMBIENTE TERAPEUTICO

In che modo curi maggiormente l'ambiente in cui si svolge il trattamento, inteso come "contesto terapeutico"?

- Design ambientale accurato (colore delle pareti, piante, quadri, poster ecc.)
- Attrezzature di ultima generazione
- Architettura adeguata al rispetto della privacy
- Comfort (illuminazione naturale, ventilazione e riscaldamento, aromi gradevoli, musica ecc.)

Sezione 9/9

CONCLUSIONI

Ritieni importante conoscere ed approfondire i fattori di contesto, durante il percorso formativo, per la pratica clinica del fisioterapista?

- Si
- No
